# Supplementary material for: Naringenin attenuates inflammation and apoptosis of osteoarthritic chondrocytes via the TLR4/TRAF6/NF-κB pathway
Source: PeerJ. 2023 Nov 8;11:e16307. doi: 10.7717/peerj.16307 (PMC10638912; doi:10.7717/peerj.16307)
Supplement: Supplemental Information 2 [file peerj-11-16307-s002.docx]

**Figure 3D Collagen II**

**
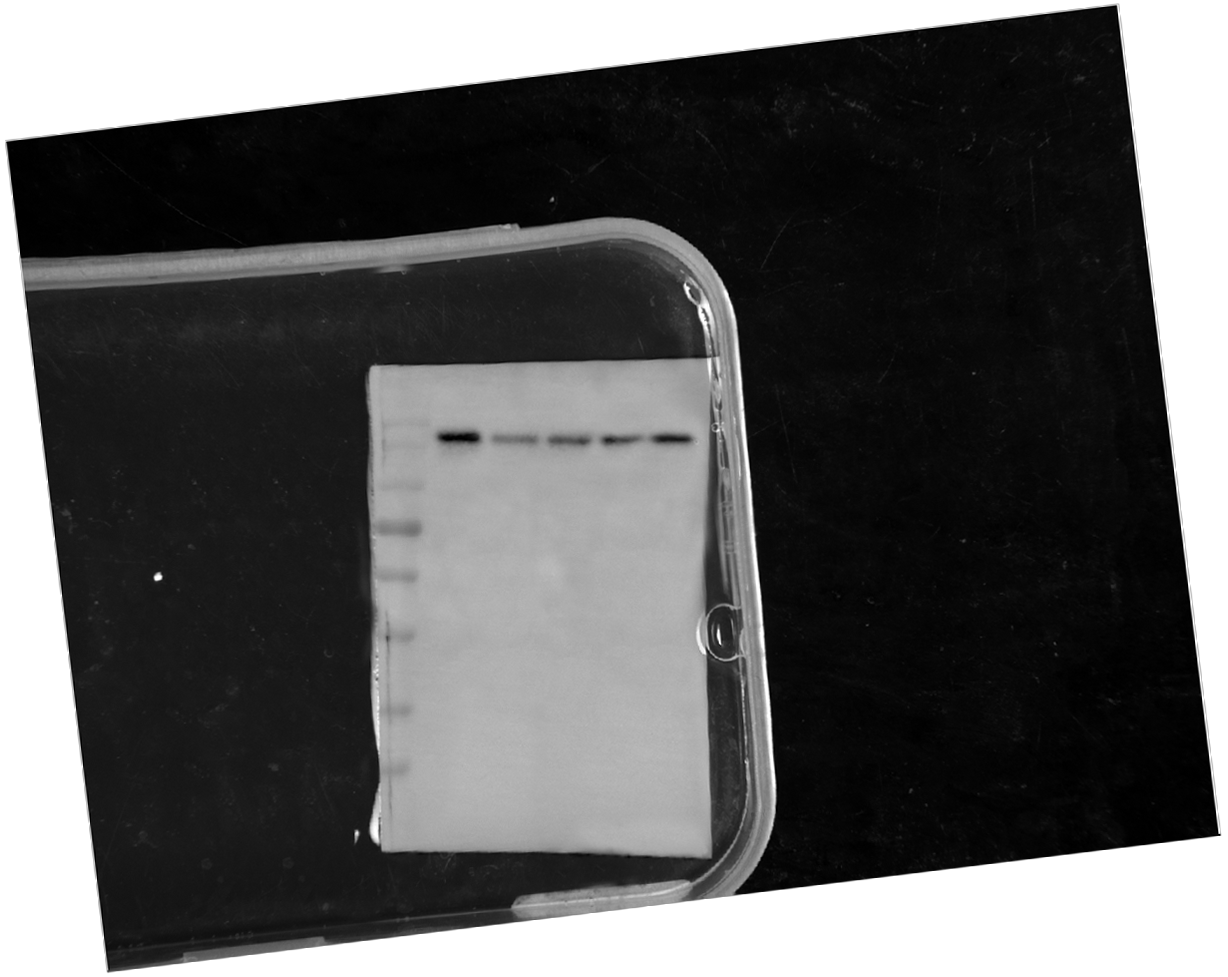
**

**Figure 3D ADAMTS5**

**
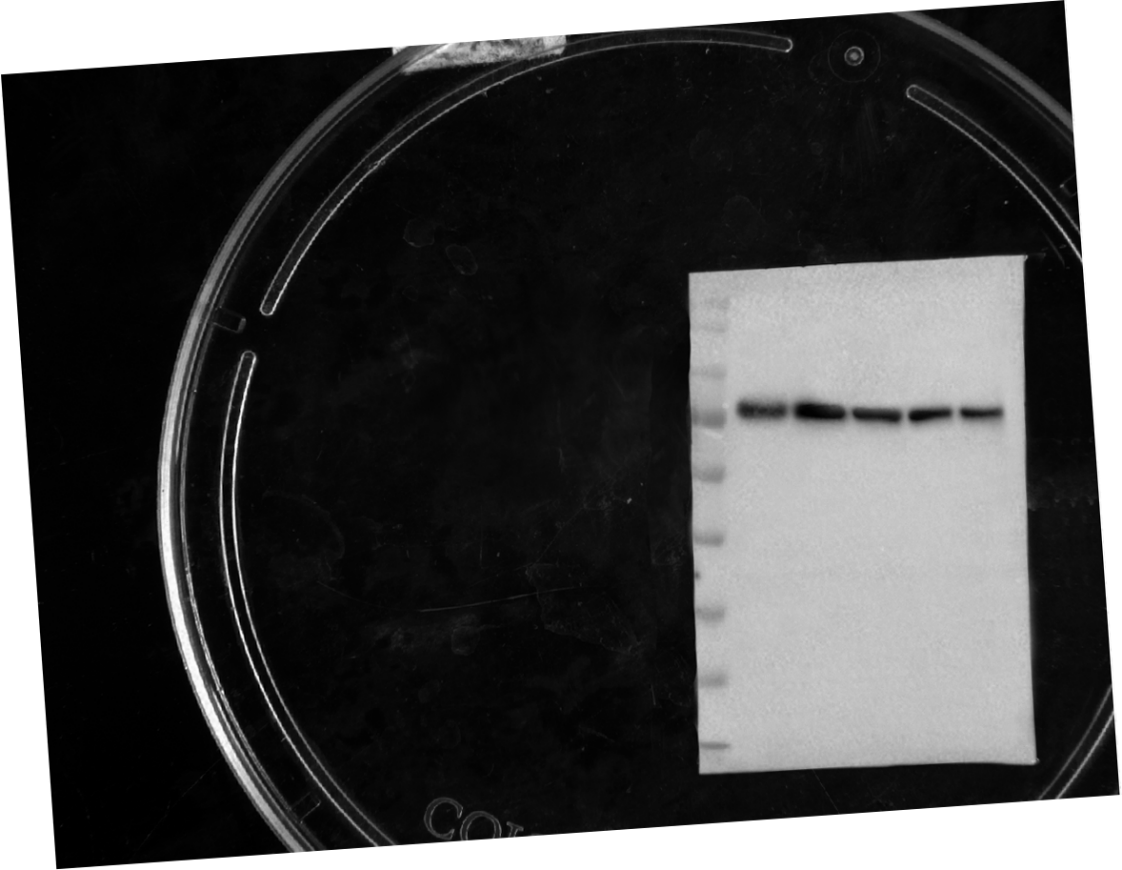
**

**Figure 3D GAPDH**

**
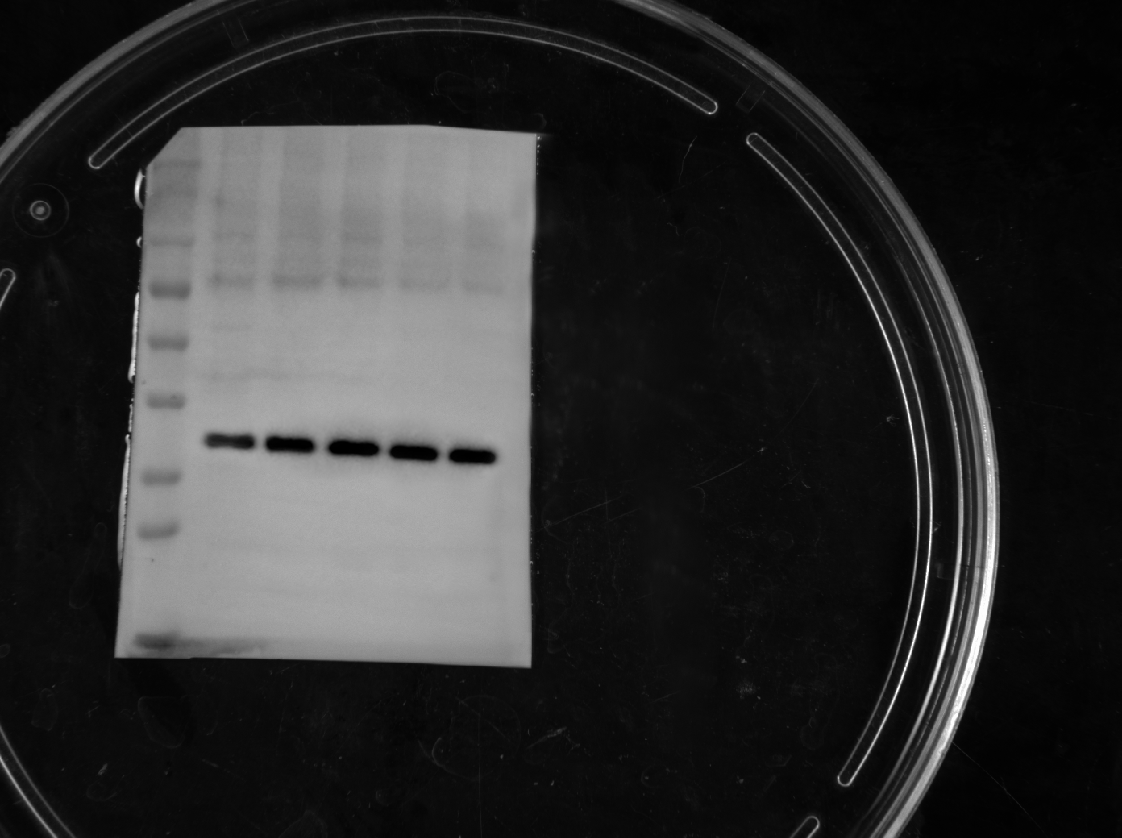
**

**Figure 4B cleaved caspase3**

**
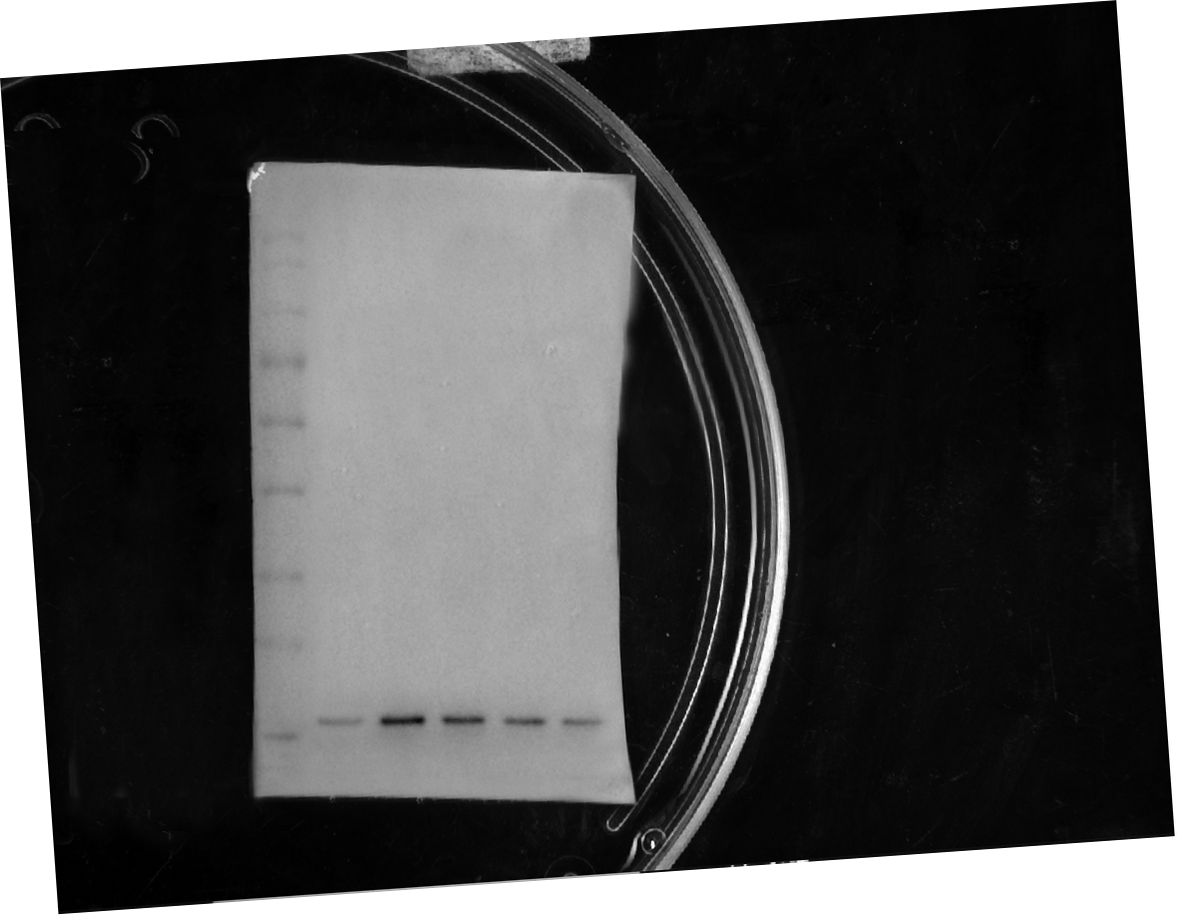
**

**Figure 4B GAPDH**

**
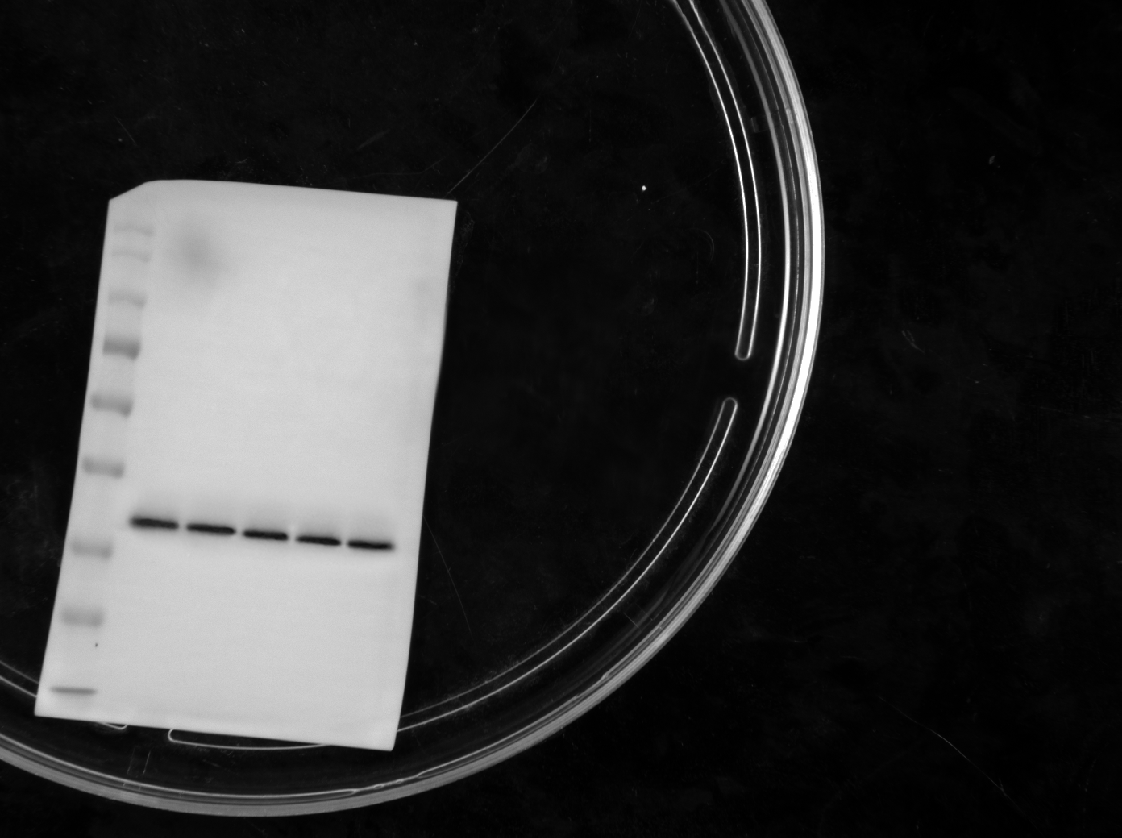
**

**Figure 5A TLR4**

**
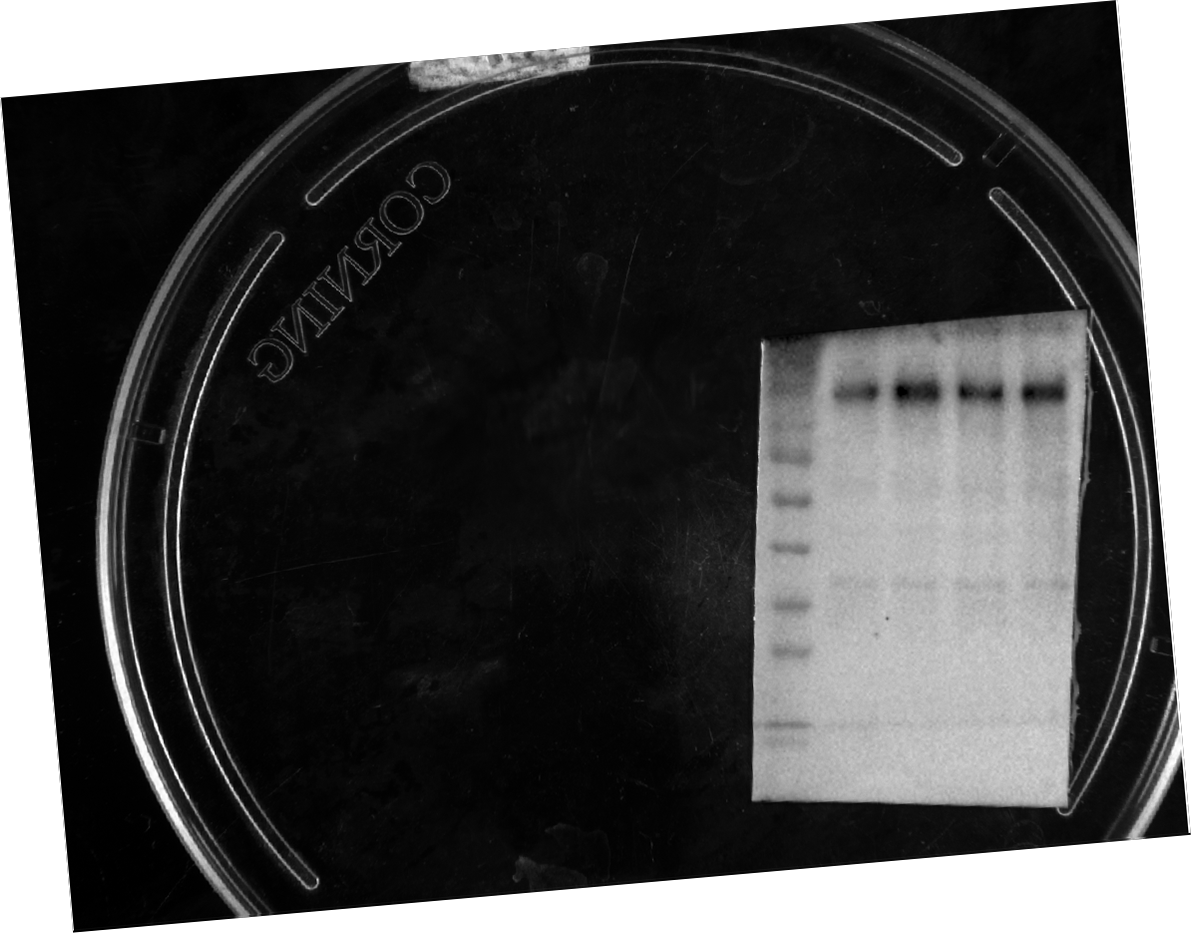
**

**Figure 5A TRAF6**

**
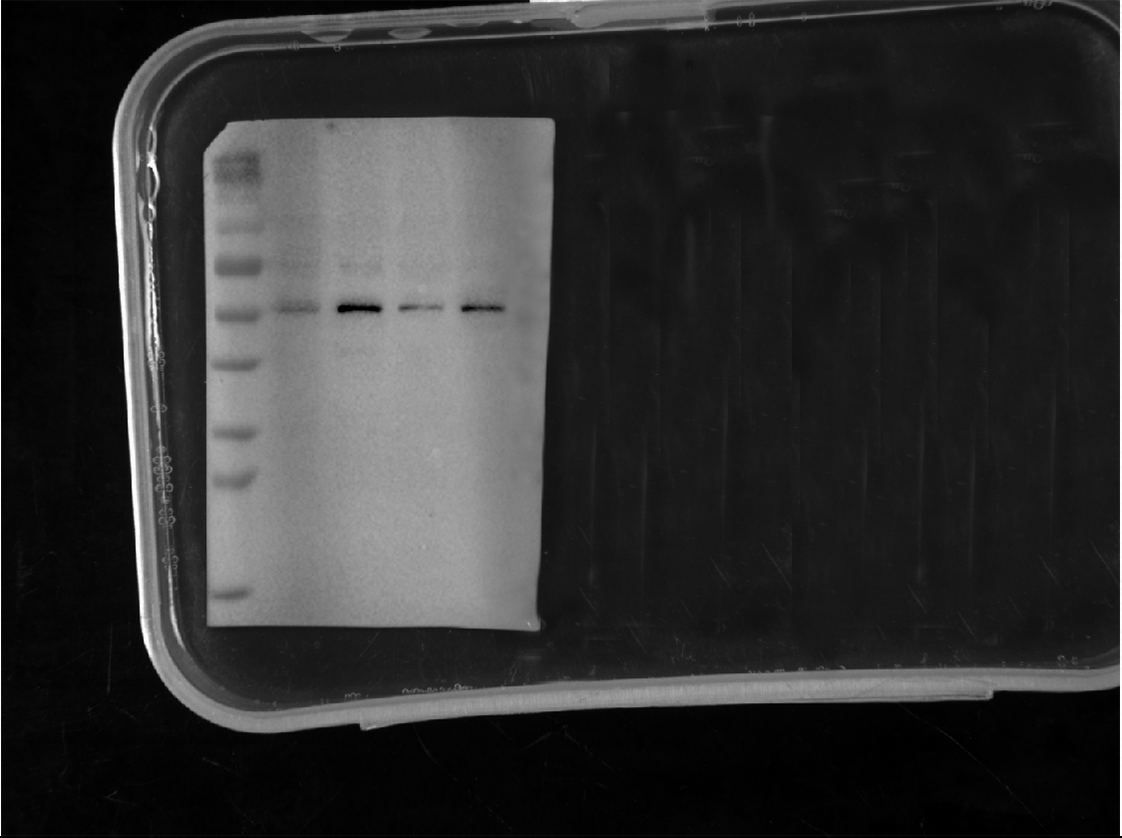
**

**Figure 5A p-NF-KB**

**
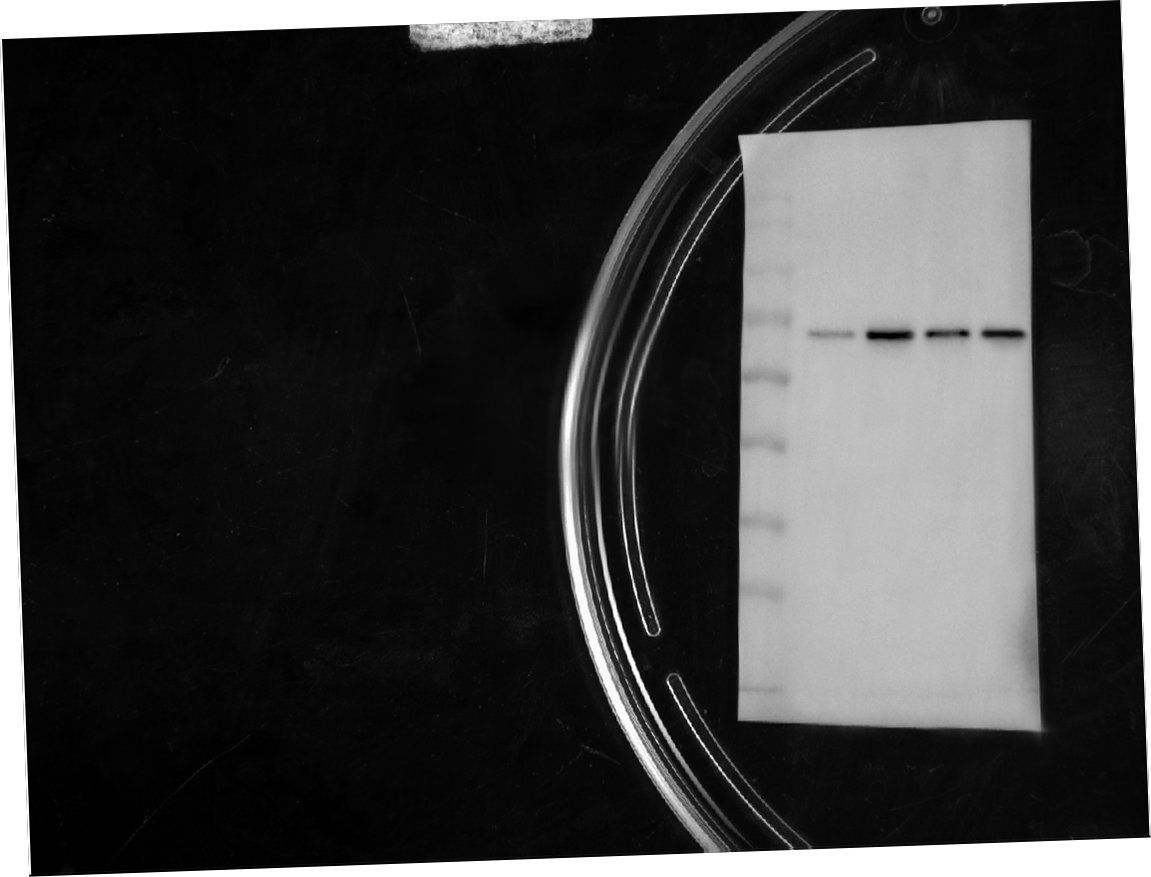
**

**Figure 5A t-NF-KB**

**
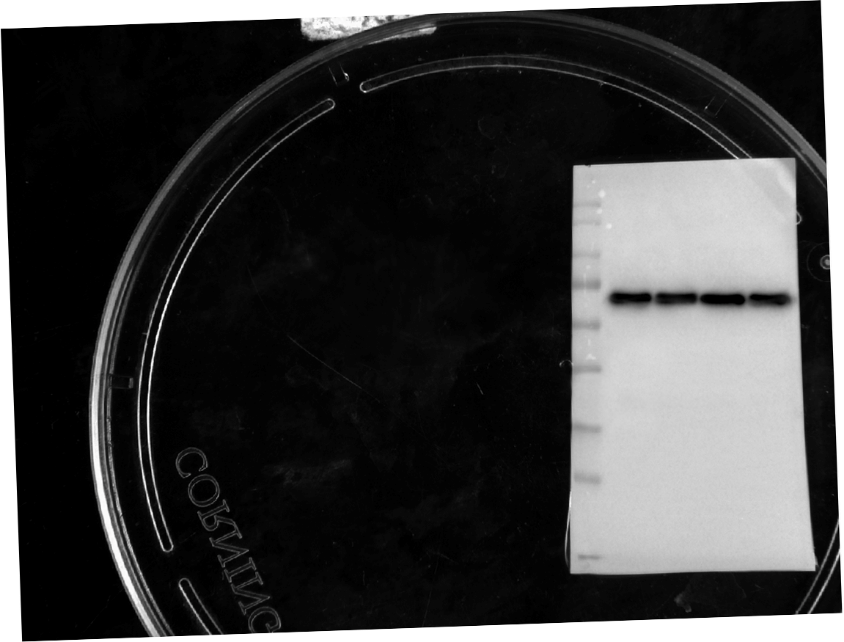
**

**Figure 5A GAPDH**

**
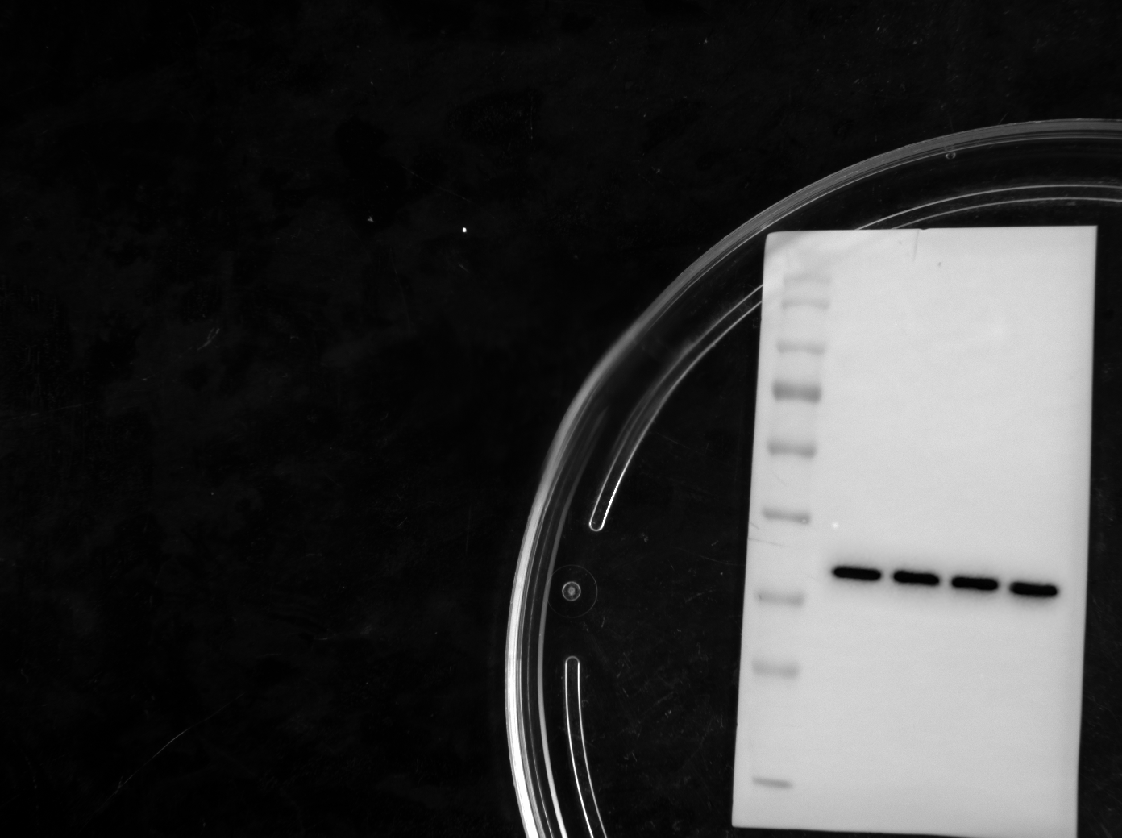
**

**Figure 6A TLR4**

**
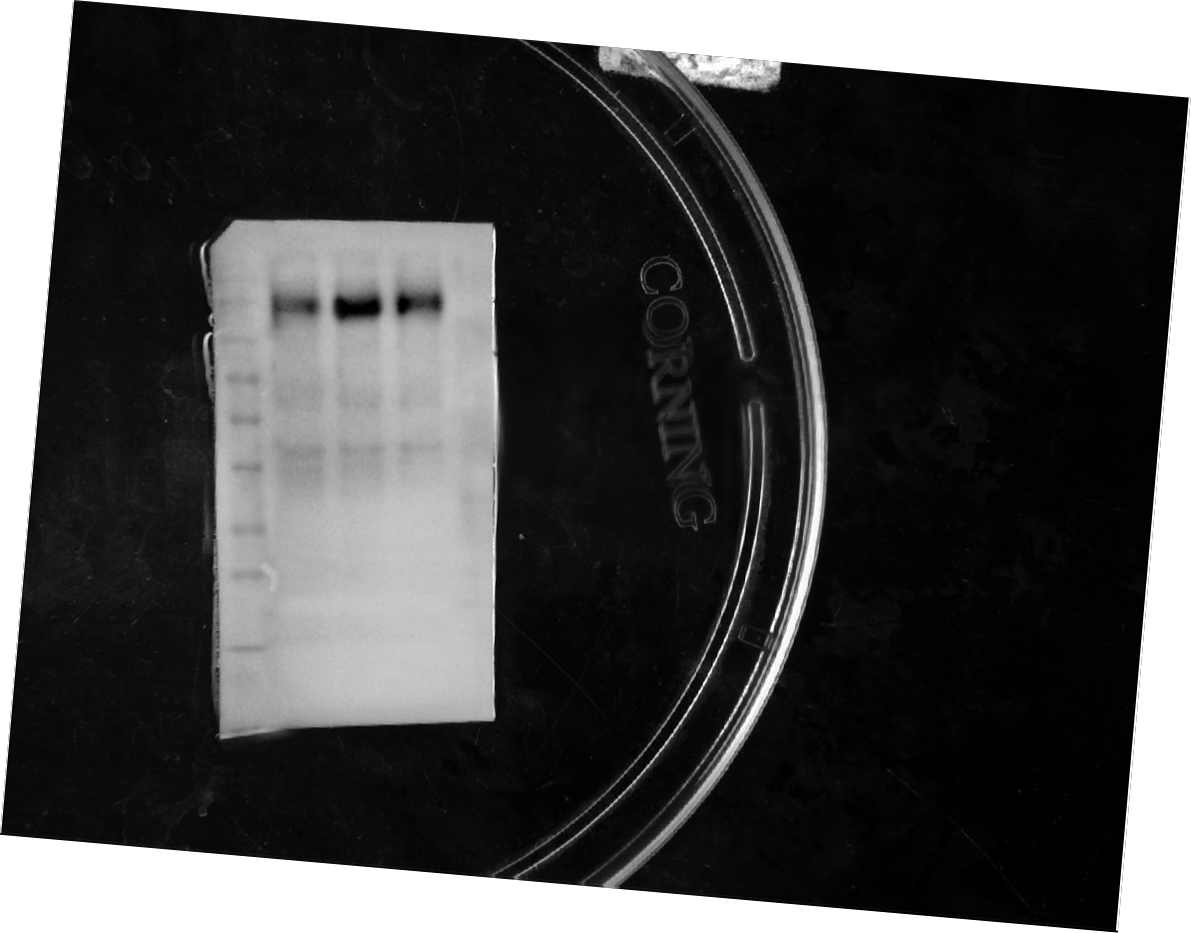
**

**Figure 6A TRAF6**

**
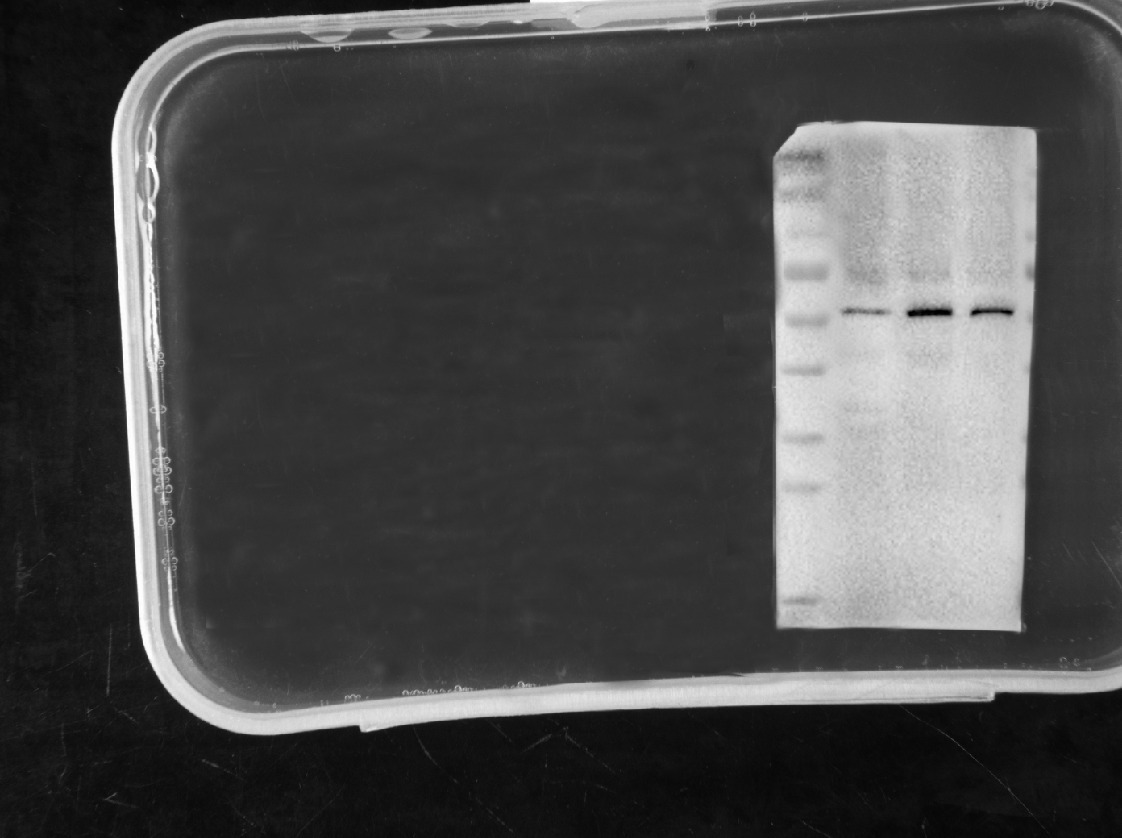
**

**Figure 6A p-NF-KB**

**
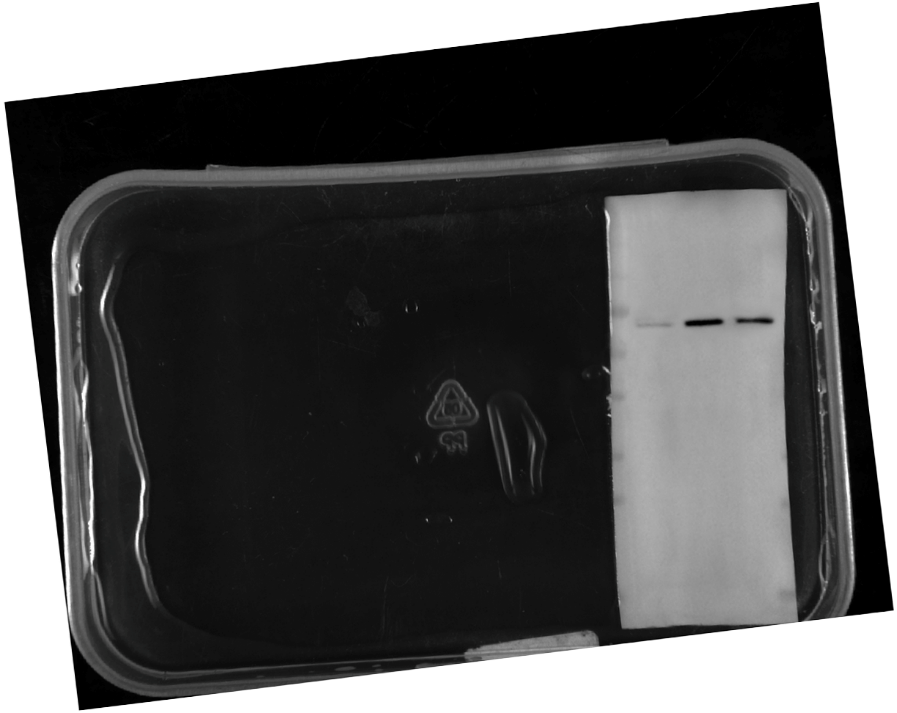
**

**Figure 6A t-NF-KB**

**
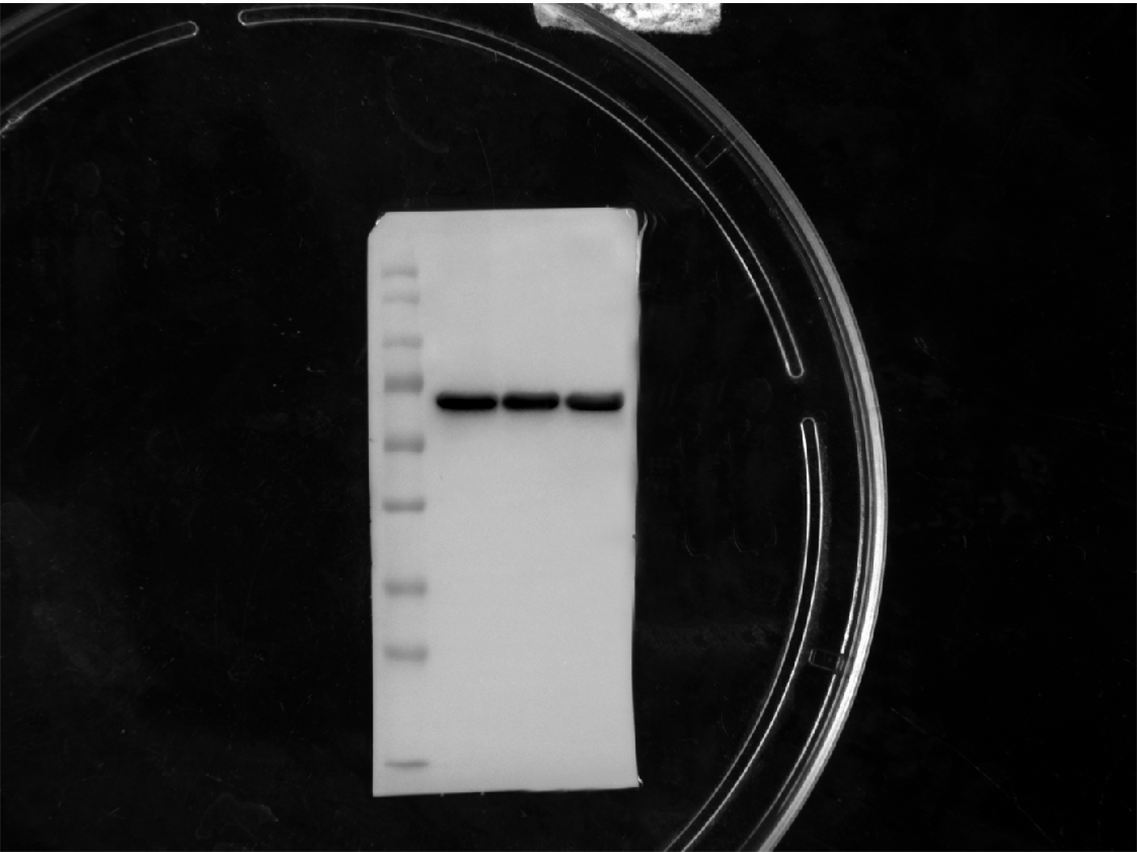
**

**Figure 6A cleaved caspase3**

**
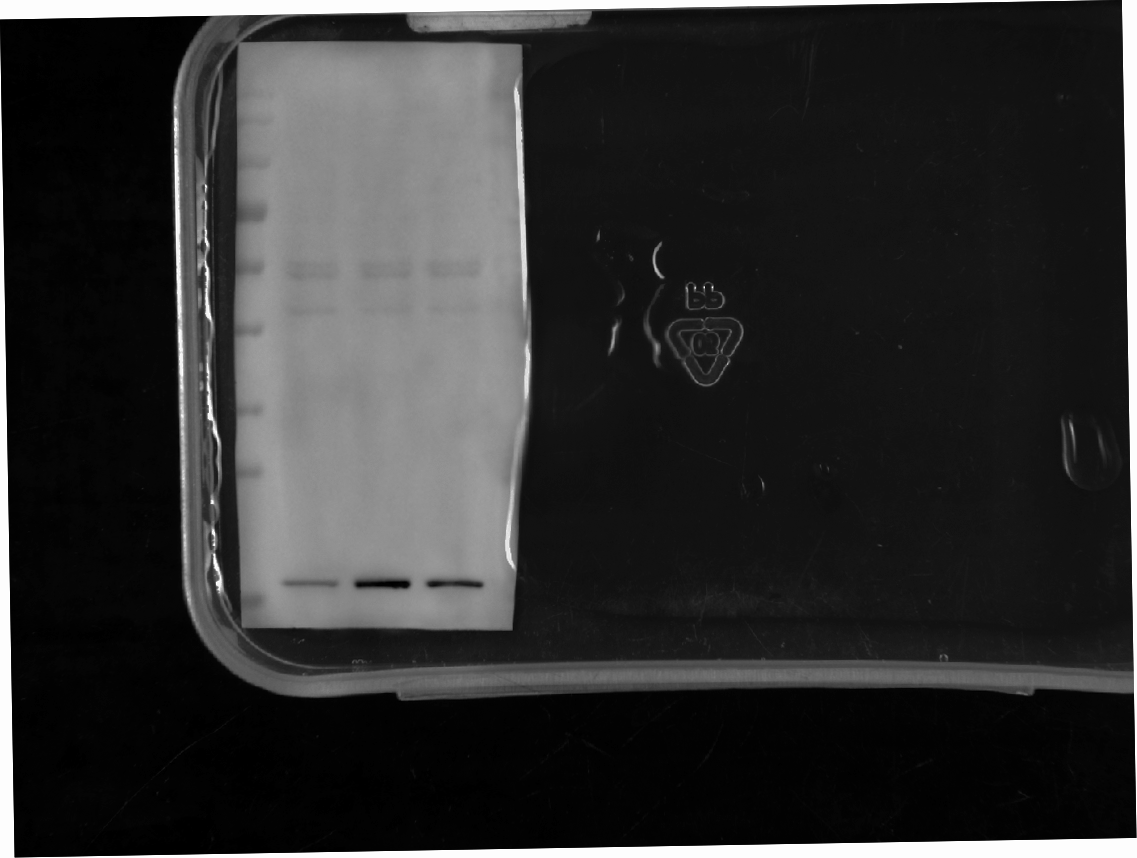
**

**Figure 6A GAPDH**

**
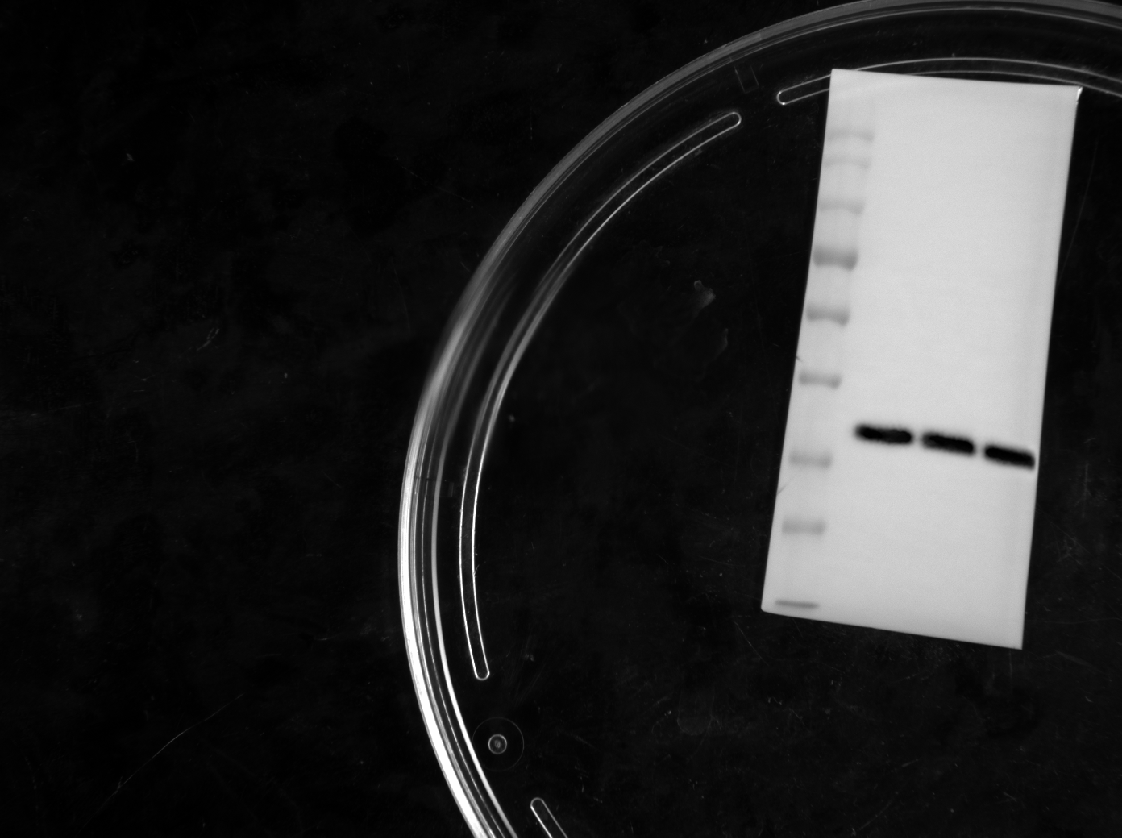
**
